# Supportive angiogenic and osteogenic differentiation of mesenchymal stromal cells and endothelial cells in monolayer and co-cultures

**DOI:** 10.1038/ijos.2016.39

**Published:** 2016-12-02

**Authors:** Florian Böhrnsen, Henning Schliephake

**Affiliations:** 1 Clinic of Oral and Maxillofacial Surgery, Georg August University, Göttingen, Germany

**Keywords:** angiogenic, co-culture, differentiation, endothelial cell, mesenchymal stromal cell, osteogenic

## Abstract

Sites of implantation with compromised biology may be unable to achieve the required level of angiogenic and osteogenic regeneration. The specific function and contribution of different cell types to the formation of prevascularized, osteogenic networks in co-culture remains unclear. To determine how bone marrow-derived mesenchymal stromal cells (BMSCs) and endothelial cells (ECs) contribute to cellular proangiogenic differentiation, we analysed the differentiation of BMSCs and ECs in standardized monolayer, Transwell and co-cultures. BMSCs were derived from the iliac bone marrow of five patients, characterized and differentiated in standardized monolayers, permeable Transwells and co-cultures with human umbilical vein ECs (HUVECs). The expression levels of CD31, von Willebrand factor, osteonectin (ON) and Runx2 were assessed by quantitative reverse transcriptase polymerase chain reaction. The protein expression of alkaline phosphatase, ON and CD31 was demonstrated *via* histochemical and immunofluorescence analysis. The results showed that BMSCs and HUVECs were able to retain their lineage-specific osteogenic and angiogenic differentiation in direct and indirect co-cultures. In addition, BMSCs demonstrated a supportive expression of angiogenic function in co-culture, while HUVEC was able to improve the expression of osteogenic marker molecules in BMSCs.

## Introduction

Sites of implantation with compromised biology may be unable to achieve the required level of osteogenic activity, regeneration and osseointegration.^1, 2^ The successful healing of bone grafts in these sites of implantation is based on the supply of oxygen and nutrients to the grafted tissue. In non-vascularized bone grafts, revascularization originating from the recipient site is the key process for bone graft survival and successful repair. This applies even more to the use of tissue-engineered bone grafts, the biological quality of which is still inferior to that of the native bone grafts. Monitoring of the integration of cancellous bone grafts using Technetium-99 (^99^Tc) bone scans has shown that rapid revascularization of large graft areas occurs during the first postoperative week.^3, 4^ As the velocity of this process is far beyond the growth rate of proliferating capillaries at the recipient site, it has been suggested that revascularization of grafted cancellous bone tissue is accomplished through the direct connection of proliferating vessels at the recipient site to existing capillary networks inside the grafts. For the use of tissue-engineered bone grafts, the generation of functional capillary networks inside the cultured constructs may thus be important to overcome the current limitations in the clinical application of bone tissue engineering.^5, 6^ The prevascularization of tissue-engineered bone grafts is therefore considered to be an essential step towards a graft structure that may enhance the early revascularization of tissue-engineered grafts.

Prevascularization is based upon the presence of an endothelial cell (EC) network containing vital mesenchymal stromal cells (MSCs) and precursor cells, which facilitate remodelling and regeneration.^5, 7^ However, EC and MSC interactions and their functions in reconstructing cellular networks are not well understood.^8, 9^ ECs have been reported to form microcapillary structures *in vitro*.^10^ These capillary-like networks express mature EC markers, such as PECAM-1 (CD31) and von Willebrand factor (vWF). To maintain and mature prevascularized networks, ECs interact with extracellular matrix components and surrounding cells.^11, 12, 13^ It has been suggested that MSCs, with their unique characteristics to represent pericyte-like features^14^ and differentiate along the osteogenic lineage,^15^ contribute to the microvessel network and facilitate prevascularized osteogenic differentiation.

Because of a high degree of diversity of *in vitro* and *in vivo* studies, the contributions of different cell types to the formation of a microcapillary network with osteogenic properties remain elusive. Although studies have demonstrated that MSC-conditioned media are able to promote EC viability,^16^ we hypothesize that the successful establishment of prevascularized networks is co-dependent on an endothelial and supporting MSC co-culture differentiation. Different types of MSCs have been characterized^17, 18, 19^; however, it remains unclear whether MSCs can retain their unique osteogenic potential during angiogenic differentiation. To determine how MSC and EC contribute to a cellular interdependent proangiogenic differentiation, we analysed the differentiation of bone marrow-derived MSCs (BMSCs) and ECs under the influence of EC growth medium in monolayers, Transwells and co-cultures.

## Materials and methods

### Isolation of BMSCs

The isolation of human BMSCs was conducted using bone marrow aspirates of five patients aged between 8 and 58 years with the patients' informed consent and according to the guidelines and approval of the local ethics committee (No. 15/10/01). None of the patients was known to have infections, cancers, chronic diseases or any generalized bone marrow or connective tissue diseases. The aspirates were obtained during the procurement of bone grafts for the augmentation of the mandible and/or maxilla. The isolation of BMSCs was performed using density gradient centrifugation for 20 min at 800*g*. The light band that formed between the lymphocyte and erythrocyte band was separated, and the cells were acquired, washed and centrifuged for 5 min at 300*g*. The number of cells was determined and the cell suspension was plated onto 75 cm^2^ tissue culture flasks. Non-adherent cells were removed by the first medium change after 24 h. Single colonies of adherent fibroblast-like cells were first visible after 72 h of culture. All cultures were performed at 37 °C and 5% CO_2_.

### Culture and characterization of BMSCs and human umbilical vein ECs

BMSCs were cultured in basal medium consisting of high-glucose Dulbecco's modified Eagle's medium (DMEM) supplemented with 1% non-essential amino acids, 0.1 mmol̇L^−1^ β-mercaptoethanol, 2% gentamicin and 10% foetal bovine serum (FBS). When adherent cells reached approximately 80%–90% confluence, they were washed with phosphate-buffered saline (PBS), trypsinized and centrifuged for 5 min at 250*g*. The cells were plated at a density of 1 × 10^4^ cells per cm^2^. Plastic adherent cell populations were homogeneous and exhibited a typical spindle-shaped morphology. The cells did not differentiate spontaneously during culture expansion into any morphologically identifiable cell type. Mesenchymal and haematopoietic cluster antigens were evaluated by means of flow cytometry as has been described previously.^20^ BMSCs were subsequently stained for CD45 phycoerythrin (PE; Clone: HI30), CD34 PE (Clone:581) to discriminate human BMSCs from cells of haematopoietic origin. In addition, CD105 PerCP-Cy5.5 (Clone: 266), CD90 fluorescein isothiocyanate (FITC; Clone: 5E10), and CD73 allophycocyanin (Clone: AD2) were included in the phenotyping profile. All monoclonal antibodies were purchased from Becton Dickinson (Heidelberg, Germany). Cells were analysed on a Cytomics FC 500 flow cytometer using Cytomics CXP software (Beckman Coulter, Krefeld, Germany). To counter interindividual differences, BMSC isolates were pooled and the resulting culture was used for further differentiation. Passages used for differentiation in three independent samples per experimental group (*n*=3) were p2, p3 and p4. Cryopreserved HUVECs were commercially obtained from Lonza Group (Basel, Switzerland) and tested for mycoplasma, bacteria, yeast, fungi, HIV-1, hepatitis B and hepatitis C by the distributor. Passages used for culture and differentiation were p2, p3 and p4. Further analysis was carried out using triplicates at minimum.

### Differentiation of HUVECs and BMSCs in monolayers, Transwells and co-cultures

**Figure 1:**
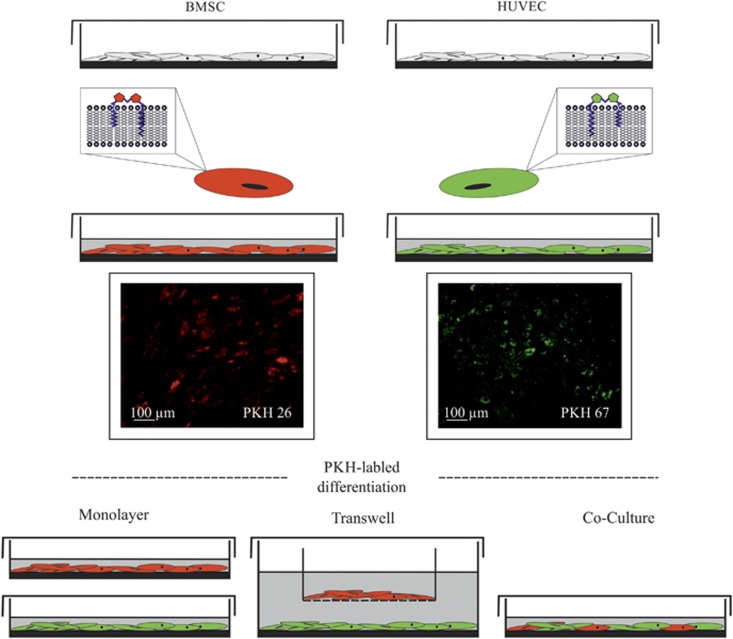
**Culture and PKH labelling of BMSCs and HUVECs for further differentiation in monolayers, Transwells and direct co-culture**. To ensure stable growth characteristics, the optimal cell growth and maintenance was achieved using a plating density of 1 × 10^4^ cells per cm^2^ and a cell ratio of BMSC:HUVEC of 1:2.5 in co-culture. BMSCs, bone marrow-derived mesenchymal stromal cells; HUVECs, human umbilical vein endothelial cells.

To assess the interdependent influence of angiogenic differentiation in BMSCs and HUVECs, both were analysed using Transwell and co-culture differentiation in comparison to monolayer culture. To ensure stable growth characteristics, optimal cell growth and maintenance was achieved using a plating density of 1 × 10^4^ cells per cm^2^ and a cell ratio of BMSC:HUVEC of 1:2.5 in co-culture (Figure 1). To induce angiogenic differentiation, cells were treated with EC growth medium EGM-2 SingleQuots (Lonza Group, Basel, Switzerland) for 10 days. EGM-2 has been optimized for EC differentiation and contains 2% FBS and vascular endothelial growth factor for rapid proliferation. Standardized media conditions were guaranteed by the manufacturer.

### PKH labelling of HUVEC and BMSC co-cultures

The PKH fluorescent cell linker allows the fluorescent labelling of live cells over an extended period of time, with no apparent toxic effects. HUVECs were labelled using PKH67 with green fluorochromes at an excitation of 490 nm and an emission of 504 nm. BMSCs were labelled with PKH26 red fluorochrome, which has an excitation at 551 nm and an emission at 567 nm (Figure 1). The linkers were physiologically stable and showed little-to-no toxic side effects on cell systems, as has been tested by the provider. Cells did retain both their biological and proliferative activity. Cells were stained using the standard protocol (Sigma-Aldrich, St Louis, MO, USA). Depending on the quantity of cells used for labelling, Diludent C and PKH67 or PKH26 were added in equivalent volumes to the cell suspension. HUVECs or BMSCs were stained for 3–4 min, and the incubation was stopped by adding an equivalent volume of FBS. Cells were washed in 10 mL DMEM supplemented with 10% FBS and replated for the following experiments in EGM-2 SingleQuots.

### Alkaline phosphatase staining of HUVEC and BMSC co-cultures

Alkaline phosphatase (AP) activity was demonstrated using the AP Staining Kit (Sigma, Munich, Germany). Prior to staining, cells were washed twice with PBS and fixed in citrate-formaldehyde (2.5 mL citrate, 6.5 mL acetone, 0.8 mL formaldehyde 37%) for 30 s. Fixation was thoroughly removed with distilled water, and the cells were incubated in naphthol stain for 15 min at room temperature according to the manufacturer's recommendations. After incubation, the staining solution was removed, and the samples were rinsed with distilled water, embedded in mounting media and stored at 4 °C.

### Fluorescent immunostaining of HUVEC and BMSC co-cultures

HUVECs and BMSCs cultured in monolayers, Transwells and co-culture were rinsed three times with PBS, fixed for 5 min with precooled (−20 °C) methanol–acetone at 4 °C, washed four times with PBS and incubated at room temperature for 30 min with 7.5% bovine serum albumin. Specimens were then incubated for 1 h with a primary antibody in a humidified chamber at 37 °C. Antibodies specific for the following proteins were used (designation, dilution ratio in PBS and references are given in parentheses): CD31 (PECAM-1; 1:50), and osteonectin (ON; AON-1; 1:20).^21^ The antibodies were obtained from the Developmental Studies Hybridoma Bank (University of Iowa, Iowa City, IA, USA). After rinsing four times with PBS, slides were incubated for 1 h at 37 °C with Alexa Fluor 594 (Life Technologies GmbH, Darmstadt, Germany) and Fluorescein isothiocyanate (FITC; Merck KGaA, Frankfurt, Germany) labelled anti-mouse IgG and 4′,6-diamidino-2-phenylindole dihydrochloride (Sigma, Taufkirchen, Germany). Slides were washed four times in PBS and briefly washed in distilled water. After immunostaining, the specimens were embedded in FluorPreserve mounting medium (Merck KGaA, Darmstadt, Germany) and analysed with the fluorescence microscope Axioskop (ZEISS, Oberkochen, Germany) or FV1000 confocal laser scanning microscope (Olympus, Tokyo, Japan). Negative controls were performed using the secondary antibody only.

### Quantitative reverse transcriptase polymerase chain reaction analysis of HUVEC and BMSC co-cultures

HUVECs and BMSCs cultured *via* monolayer, indirect and direct co-culture were collected after 10 days of differentiation, washed twice with PBS and total RNA was isolated using a standardized RNA Isolation Kit (RNeasy Mini Kit; Quiagen AG, Hombrechtikon, Switzerland) according to the manufacturer's recommendations. Samples were treated with DNAse-I to remove genomic DNA contamination. Samples were precipitated, washed in 75% ethanol, resuspended in 50 μL RNase-free water and stored at −80 °C. RNA quality was determined by the use of microfluidic electrophoresis (Agilent 2100 Bioanalyzer; Agilent Technologies, Santa Clara, CA, USA). The RNA concentrations were determined by measuring the absorbance at 260 and 280 nm. Samples of 200 ng RNA were reverse transcribed using the iScript cDNA Synthesis Kit (Bio-Rad Laboratories, Hercules, CA, USA). Aliquots of 5 μL from the reverse transcriptase reactions were used for the amplification of transcripts using primers specific for CD31, vWF, ON, Runx2 and β-actin (Table 1). All samples were stored at −80 °C for further analysis. For relative cDNA quantification, the Bio-Rad MyIQ real-time polymerase chain reaction (PCR) detection system with the Bio-Rad iQ SYBR Green Supermix was used. PCR quantification was carried out after denaturation for 30 s at 98 °C followed by amplification and measurement for 45 cycles of 1 s denaturation at 94 °C, 15 s annealing at 60 °C and 10 s elongation at 72 °C.

### Statistical analysis

Statistical analysis was performed using the GraphPadPrism 5.0 software (GraphPad Software, La Jolla, CA, USA). For quantitative reverse transcriptase polymerase chain reaction (qRT-PCR), relative expression ratios were determined using the Δct mathematical model for relative quantification. After log_2_ transformation, differences in gene expression were identified by Kruskal–Wallis analysis. Additional nonparametric testing of vWF and ON was performed using Mann–Whitney analysis. Samples were analysed at least in three independent experiments (*n*=3) using triplicates at minimum. The level of significance was set to 5%.

### Ethical standards

The isolation of human BMSCs was carried out in accordance with the patients' informed consent and according to the guidelines and approval of the local ethics committee (No. 15/10/01) of the Georg August University, Göttingen, Germany. It has therefore been performed in accordance with the ethical standards established in the 1964 Declaration of Helsinki and its later amendments.

## Results

### Immunophenotypic characterization of isolated BMSCs

Isolated BMSCs were negative for CD45 and CD34 and stained positive for CD105, CD90 and CD73. The isolated cells showed a distinct phenotypic population (>90% homogeneous in passage 2). The stem/progenitor cell isolates were negative for CD45 (leukocyte common antigen) and CD34 (gp105-120), which indicated that they were not of haematopoietic origin. The cells expressed ecto-5′-nucleotidase (CD73) and CD90 (thymocyte differentiation antigen-1, Thy-1) and matrix receptor CD105 (endoglin, SH2).

### Angiogenetic differentiation of HUVEC and BMSC co-cultures

**Figure 2:**
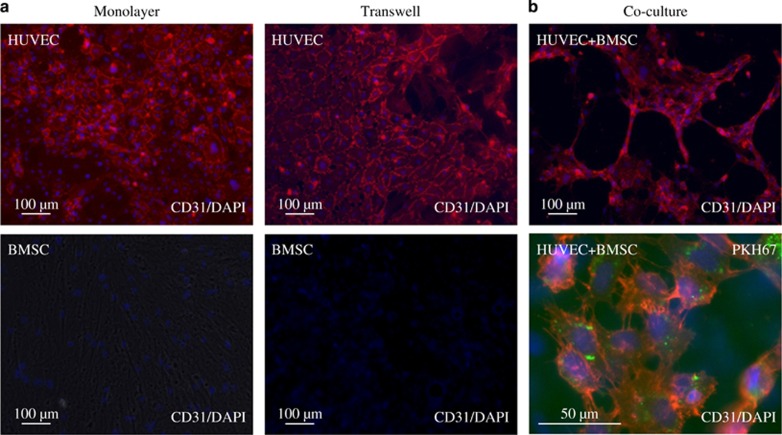
**The expression of the angiogenic marker molecule CD31 in monolayer, Transwell and co-culture differentiation**. (**a**) CD31 (red) was detected after 10 days of differentiation in HUVECs. No CD31 expression was observed in BMSCs during monolayer and Transwell cultures by immunostaining. (**b**) Co-cultured BMSCs and HUVECs arranged into clusters. Double fluorescence staining showed that CD31 associated with PKH67 (green)-labelled HUVECs. Samples were analysed at least in three independent experiments (*n*=3) using triplicates at minimum. BMSCs, bone marrow-derived mesenchymal stromal cells; DAPI, 4′,6-diamidino-2-phenylindole dihydrochloride; HUVECs, human umbilical vein endothelial cells.

Immunostaining revealed the expression of the angiogenic marker molecule CD31 in monolayer, Transwell and co-culture differentiation (Figure 2a and 2b). CD31 was strongly detected after 10 days of differentiation in HUVECs. No CD31 expression was observed in BMSCs during monolayer and Transwell cultures by immunostaining. In addition, co-culture differentiation demonstrated structural changes compared with monolayer and Transwell cultures. Monolayer and Transwell cultures showed a homogeneous single-cell layer arrangement (Figure 2a). However, co-cultured MSCs and HUVECs arranged into clusters bridged by additional interconnecting networks of elongated CD31-positive cells. In addition, double fluorescence staining showed that CD31 staining was associated with PKH67-labelled HUVECs (Figure 2b).

**Figure 3:**
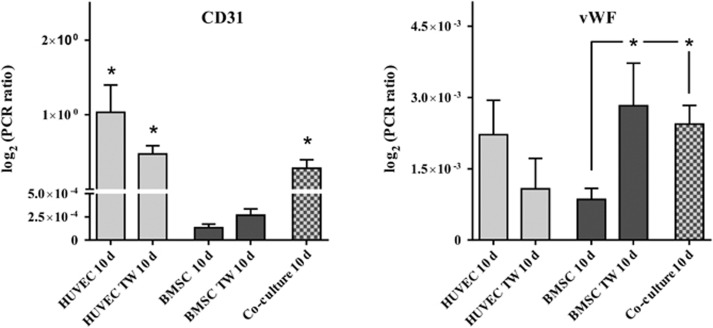
**qRT-PCR analysis of CD31 and vWF expression in HUVEC and BMSC culture**. The results of monolayer (HUVEC/BMSC), Transwell and co-culture are shown. qRT-PCR analysis demonstrated the significant expression of the angiogenic marker molecule CD31 in both monolayer and Transwell cultures of HUVECs (*P*<0.05). BMSCs failed to express high levels of CD31 in comparison to HUVEC cultures. Samples were analysed at least in three independent experiments (*n*=3) using triplicates at minimum. *P<0.05. BMSCs, bone marrow-derived mesenchymal stromal cells; HUVECs, human umbilical vein endothelial cells; qRT-PCR, quantitative reverse transcriptase polymerase chain reaction; TW, transwell; vWF, von Willebrand factor.

qRT-PCR analysis demonstrated a significant expression of the angiogenic marker molecule CD31 in both monolayer and Transwell cultures of HUVECs (*P*<0.05; Figure 3). BMSC failed to express high levels of CD31 in comparison to HUVEC cell cultures. However, associated with the expression of CD31 in HUVEC, co-cultures of BMSCs and HUVECs demonstrated a significant expression of CD31 (*P*<0.05) and reached a level of CD31 expression equivalent to that found in HUVEC cultures. Although monolayer cultures of BMSCs revealed a reduced level of vWF, combining BMSCs with HUVECs in Transwell culture led to an improved vWF expression in BMSCs, which was also found in co-cultures (*P*<0.05; Figure 3).

### Osteogenic differentiation of HUVEC and BMSC co-cultures

**Figure 4:**
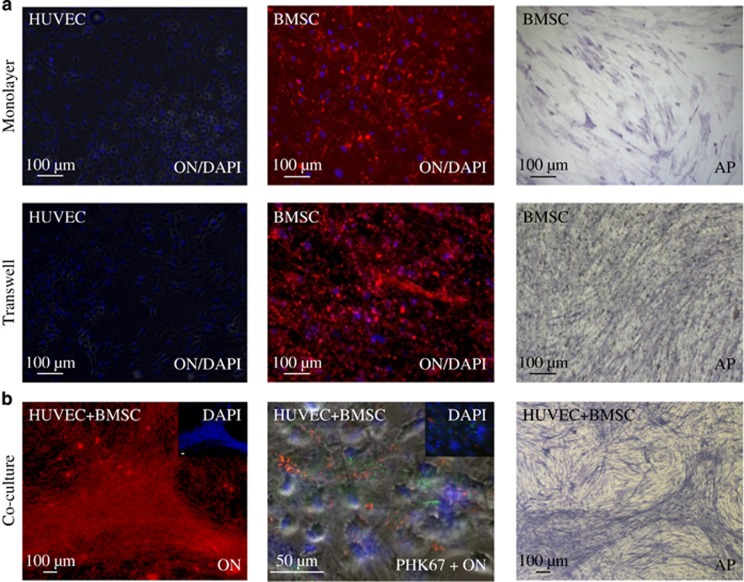
**The expression of the osteogenic marker molecules ON and AP in monolayer, Transwell and co-culture differentiation**. Immunostaining revealed the expression of ON in all BMSCs after 10 days of differentiation in monolayer, Transwell and co-culture differentiation. (**a**) HUVECs did not reveal any expression of ON in monolayers of Transwell culture. BMSC monolayer and Transwell cultures revealed homogenous staining of AP during differentiation. (**b**) Co-cultures displayed an increased level of structural organization with cells staining positive for ON and AP grouped in clusters. Double fluorescence staining revealed ON (green) to be associated with PKH26 (red)-labelled BMSC. Samples were analysed at least in three independent experiments (*n*=3) using triplicates at minimum. AP, alkaline phosphatase; BMSCs, bone marrow-derived mesenchymal stromal cells; DAPI, 4′,6-diamidino-2-phenylindole dihydrochloride; HUVECs, human umbilical vein endothelial cells; ON, osteonectin.

Immunostaining revealed the expression of the osteogenic marker molecule ON in all BMSCs after 10 days of differentiation in monolayer, Transwell and co-culture differentiation (Figure 4a and 4b). HUVECs did not reveal any expression of ON in monolayer or Transwell culture (Figure 4a). BMSC monolayer and Transwell cultures revealed a homogenous staining of AP during differentiation. In addition, co-culture differentiation demonstrated an increased level of structural organization compared with monolayer and Transwell cultures. Here cells staining positive for ON and AP grouped in interconnected clusters. Double fluorescence staining revealed ON to be associated with PKH26-labelled BMSCs (Figure 4b).

**Figure 5:**
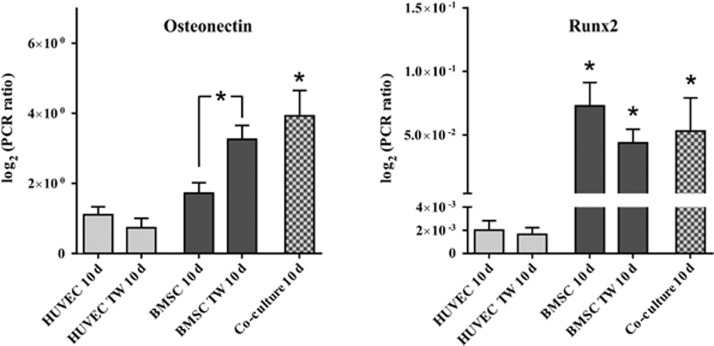
**PCR analysis of osteonectin and Runx2 expression in HUVEC and BMSC cultures**. The results of monolayer (HUVEC/BMSC), Transwell and co-culture are shown. BMSCs showed a strong expression of osteonectin, which significantly increased in Transwell culture in comparison to monolayer culture (*P*<0.05). The highest expression of osteonectin was found under co-culture conditions (*P*<0.05). Samples were analysed at least in three independent experiments (*n*=3) using triplicates at minimum. *P<0.05. BMSCs, bone marrow-derived mesenchymal stromal cells; HUVECs, human umbilical vein endothelial cells; qRT-PCR, quantitative reverse transcriptase polymerase chain reaction; TW, transwell.

qRT-RCR analysis confirmed the differentiation and expression of the osteogenic marker molecules ON and Runx2 (Figure 5). BMSCs showed a strong expression of ON, which significantly increased in Transwell culture in comparison to monolayer culture (*P*<0.05). The highest expression of ON was found under co-culture conditions (*P*<0.05). Analysing the expression of Runx2 in monolayer, Transwell and direct co-culture, BMSCs demonstrated a significantly higher expression in comparison to HUVEC cultures (*P*<0.05). This high level of Runx2 expression was also observed in direct co-culture (*P*<0.05).

### Morphology of HUVEC and BMSC co-cultures

**Figure 6:**
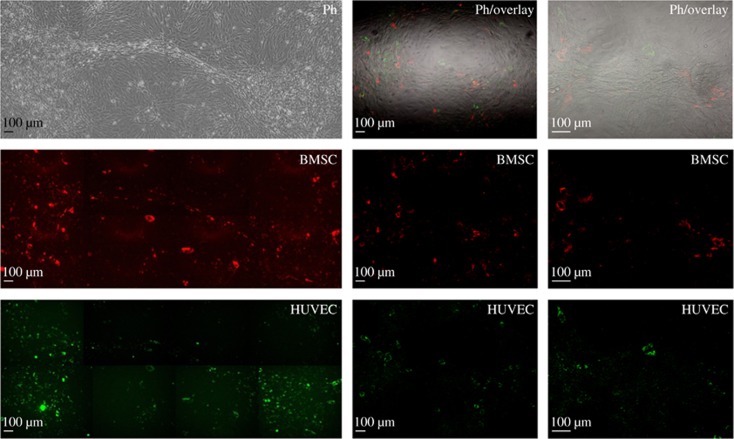
**Live cell imaging of PKH-labelled cells during MSC and HUVEC co-culture after 7 days of co-culture**. BMSCs are labelled with PKH26 red fluorochrome. HUVECs are labelled using PKH67 with green fluorochromes. After 1 week of angiogenic co-culture, both cell lines were often found in clusters with additional interconnecting networks. BMSCs, bone marrow-derived mesenchymal stromal cells; HUVECs, human umbilical vein endothelial cells; MSC, mesenchymal stromal cell.

To further study the morphological changes in the cellular arrangement of BMSCs and HUVECs in direct co-culture, cells were marked using PKH fluorescent membrane labelling. HUVECs were labelled using PKH67 with green fluorochromes. BMSCs were labelled with PKH26 red fluorochrome. After 1 week of angiogenic co-culture, both cell lines were often found in clusters with additional interconnecting networks (Figure 6). This mirrored the cellular rearrangement found during immunofluorescence analysis. PKH labelling allowed for a stable *in vitro* labelling during 7–10 days of cell culture. Because of cell doubling, however, the intensity of live cell imaging is reduced over time.

## Discussion

Bioengineered bone tissue depends on a mature vascular network to deliver angiogenic and growth factors, enhance proliferation and meet metabolic demands.^22^ This vascular assembly is necessary to allow growth beyond the oxygen diffusion limit.^5, 23^ The development of a mature and functional vasculature depends on the interaction of EC with perivascular stromal cells.^22^ The ability to promote angiogenesis while maintaining the osteogenic potential of the transplant is essential to enable prevascularized bone tissue engineering. However, our understanding of angiogenic and osteogenic networks is still sparse. Strategies for osteogenic differentiation and vascularization often involve sophisticated scaffolds and the delivery of growth factors to improve differentiation.^5, 24, 25^

ECs have been demonstrated to form microcapillary structures *in vitro*.^10^ These capillary-like networks express mature EC markers such as PECAM-1 (CD31) and vWF. In addition, MSCs are known to influence and support angiogenic differentiation.^26^ Although MSC are generally considered to be negative for CD31,^27^ there is continuing evidence that MSCs harbour heterogeneous subpopulations of cells positive for PECAM-1.^28, 29^ In our study, immunostaining revealed the expression of the CD31 in all ECs analysed in monolayers, Transwells and co-culture. qRT-PCR analysis confirmed angiogenic differentiation and a high rate of CD31 expression in HUVECs correlating to the expression of the CD31 protein in co-culture (*P*<0.05). qRT-PCR revealed only marginal expression levels of CD31 in BMSC, which could not be confirmed by immunofluorescence. It has been reported that MSCs display an increased level of PECAM-1 in co-cultures with blood mononuclear cells.^30^ Because our results indicated no significant expression of CD31 in BMSCs, the co-culture expression of PECAM-1 is likely to be limited to HUVECs only. In addition, double fluorescence staining showed CD31 to be associated with PKH67-labelled HUVECs. However, previous studies have demonstrated that proangiogenic conditions are able to differentiate MSCs towards an angiogenic phenotype.^31^ Again, in our study, monolayer cultures of BMSCs differentiated under the influence of EC growth medium displayed a significantly reduced level of vWF (*P*<0.05). However, this expression was improved in BMSCs during endothelial Transwell and direct co-culture, stressing the importance of cellular dependence during differentiation, as has been suggested previously.^32^ It is therefore safe to assume that BMSCs were able to contribute to the specific angiogenic differentiation in direct co-culture under the influence of EC growth medium.

Endothelial and tubular networks formed by monocultures of HUVECs are not stable.^5^ To maintain and mature prevascularized networks, ECs interact with extracellular matrix components and surrounding cells.^11, 12^ It has been suggested that, with their unique ability to represent pericyte-like features,^5, 7, 14^ MSCs contribute to this network. However, the underlying mechanism remains unknown.^7, 33^ In our study, direct co-culture of ECs with BMSCs demonstrated a structured cellular arrangement.

MSCs have been used in the repair and regeneration of a variety of mesenchymal tissues, such as cartilage and bone.^15^ They represent a class of adult progenitor cells capable of differentiating into several mesenchymal lineages and have been isolated from a variety of tissues.^34, 35, 36^ The ability of MSCs to influence angiogenic and osteogenic differentiation and their abundant sources of origin offer a unique solution for prevascularized bone tissue engineering.^36, 37^ Because of the diversity of studies, however, the contribution of individual cell types to differentiation is rarely analysed.^22, 38^ It has been suggested that HUVECs can inhibit osteogenic MSC differentiation *in vitro*,^39^ while other studies reported a beneficial influence of ECs on osteogenic differentiation.^40, 41^ To determine whether the osteogenic differentiation of BMSCs is altered under the influence of endothelial differentiation, we compared the co-cultured BMSCs/HUVECs to those cultured in monolayer or Transwell cultures. secreted protein acidic rich in cysteine (SPARC)/ON, a matrix cellular protein that functions to regulate cell–matrix interactions, is secreted by a variety of cells and is a characteristic of osteogenesis and tissues undergoing remodelling and repair.^42, 43^ In all BMSCs, immunostaining revealed the protein expression of the osteogenic marker ON in monolayer, Transwell and co-culture differentiation. The expression of ON has been reported in ECs as a response to injuries, regulating endothelial barrier function and inhibiting growth activity.^42, 44^ Immunofluorescence analyses, however, did not reveal any expression of ON in HUVECs. It was observed in BMSCs only and is therefore likely to be limited to BMSCs in co-culture. This is supported by double fluorescence staining, which revealed ON-positive cells to be associated with PKH26-labelled BMSCs. qRT-PCR analysis confirmed the osteogenic potential and expression of Runx2 and ON in BMSCs. The expression of Runx2 and ON is not exclusive to MSCs and has been demonstrated for ECs as well.^45, 46, 47^ However, the expression of ON and Runx2 was significantly higher in BMSCs, stressing their effect on osteogenic differentiation in direct co-culture.

Taken together, our results demonstrate that BMSCs were able to retain their osteogenic potential under the influence of angiogenic Transwell and direct co-culture differentiation. In addition, Transwell co-cultures of BMSCs demonstrated an improved expression of the angiogenic marker vWF. Interestingly, we also observed and improved the expression of ON in BMSC/HUVEC Transwell co-cultures compared with monolayers, which supports the beneficial influence of ECs on osteogenic and angiogenic differentiation. To improve the future application and integration of bioengineered bone tissue at the compromised site of implantation, this beneficial interaction of MSCs and ECs demonstrates an important characteristic of prevascularized bone regeneration.

## Figures and Tables

**Table 1:** Primers used during qRT-PCR

Gene	Primer 5′–3′	Accession number
Osteonectin (hSPARC)	F: 5′-AGAGGAAACCGAAGAGGAGG-3′ R: 5′-GGCAAAGAATGTGCAGGAAG-3′	NM_003118.3
Runx2	F: 5′-TGCCTAGGCGCATTTCAGGTG-3′ R: 5′-GGCTTTGGGAAGAGCCGGGG-3′	NM_001015051.3
CD31 (PECAM1)	F: 5′-GCTGAGTCTCACAAAGATCTAGGA-3′ R: 5′- ATCTGCTTTCCACGGCATCA-3′	NM_000442.491
vWF	F: 5′-GCTGCTGGACACAAGTTTGA-3′ R: 5′- ACTCATGGGGCTCTGCATAC-3'	NM_014622.4
β-Actin	F: 5′-CTGGCACCCAGCACAATG-3′ R: 5′-CCGATCCACACGGAGTACTTG-3′	NM_001101.3

F, forward; qRT-PCR, quantitative reverse transcriptase polymerase chain reaction; R, reverse; vWF, von Willebrand factor.
